# A 31-Year-Old Female With Postcoital Hemoptysis

**DOI:** 10.7759/cureus.48356

**Published:** 2023-11-06

**Authors:** Dawood Shehzad, Humna Younis, Mustafa Shehzad, Noor Zara, Muhammad Ahmad

**Affiliations:** 1 Internal Medicine, Holy Family Hospital, Rawalpindi, PAK; 2 Internal Medicine, Hackensack University Medical Center, Hackensack, USA; 3 Internal Medicine, Shifa Tameer-E-Millat University, Shifa College of Medicine, Islamabad, PAK; 4 Department of Surgery, Khyber Medical University, Peshawar, PAK

**Keywords:** pulmonary congestion, hypertension, mitral regurgitation, hemoptysis, postcoital

## Abstract

Hemoptysis is a common presenting symptom in the clinical setting. The most frequent causes of non-life-threatening hemoptysis, which usually originates from the pulmonary arterial circulation, include pulmonary pathologies such as infections, inflammatory airway diseases, and bronchial neoplasms. Hemoptysis occurring only after sexual intercourse, however, is a rare phenomenon. Most of the reported cases have resulted from an underlying cardiac pathology that is predisposed to acute cardiovascular decompensation, a sharp increase in pulmonary capillary pressures, and pulmonary capillary rupture during intercourse. We present the case of a 31-year-old African-American hypertensive female who presented with a six-month history of recurrent post-coital hemoptysis. Other strenuous physical activities did not result in similar episodes. Her workup ruled out the more common etiologies of hemoptysis. Computed tomography of the chest revealed bilateral centrilobular ground-glass opacities and transthoracic and transesophageal echocardiograms revealed moderate to severe mitral regurgitation with a normal left ventricular ejection fraction of 60-65%. After management with lisinopril, she reported no new episodes of post-coital hemoptysis, and a repeat chest CT showed complete resolution of the ground glass opacities bilaterally. This underscores the importance of performing a thorough cardiovascular workup in patients presenting with only post-coital hemoptysis.

## Introduction

Hemoptysis is a phenomenon that is seen in a wide variety of conditions. It can be seen with various underlying pulmonary, infectious, hematologic, and cardiovascular disorders [1]. Hemoptysis following coitus is a rare phenomenon that has been reported in 14 published cases to date. We present the case of a 31-year-old female with episodic postcoital hemoptysis.

## Case presentation

**Figure 1:**
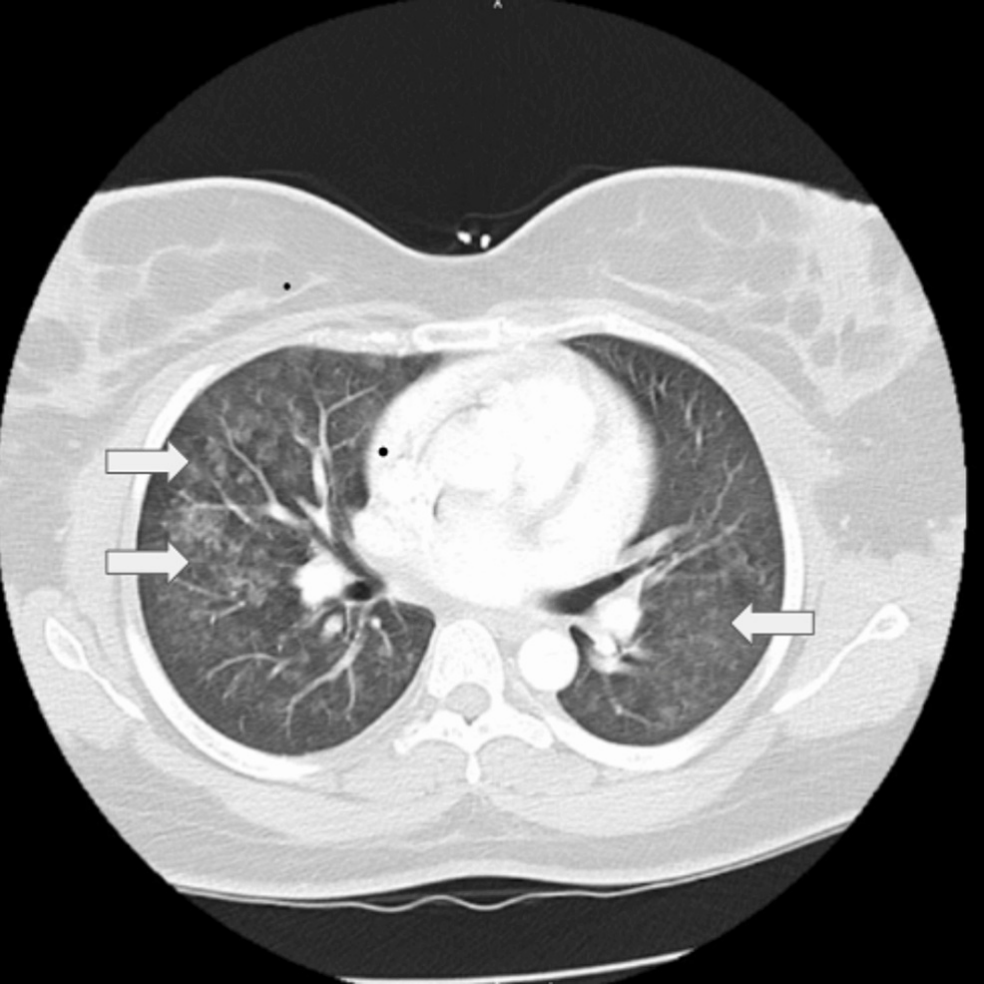
Computed tomography of the chest revealing bilateral diffuse centrilobular nodular ground-glass opacities

A 31-year-old African-American woman with a history of essential hypertension and migraines presents to the office for evaluation of recurrent episodes of hemoptysis over the last five to six months. Her most recent episode was evaluated in the local emergency room (ER). Symptoms on presentation included postcoital shortness of breath and hemoptysis. Her emergency room evaluation was significant for systolic blood pressure over 190 mmHg. A computed tomography scan (CT scan) of her chest with contrast showed bilateral centrilobular nodular ground-glass infiltrates (Figure 1). An echocardiogram was also performed during the ER evaluation and revealed a normal ejection fraction of 60-65%. The report mentioned posteriorly directed mild mitral regurgitation (MR) with an MR maximum velocity of 5.68 m/s. There was mild concentric left ventricular hypertrophy, and a thickened mitral valve was noticed, while the tricuspid valve was not well visualized. She recovered from that episode.

**Figure 2:**
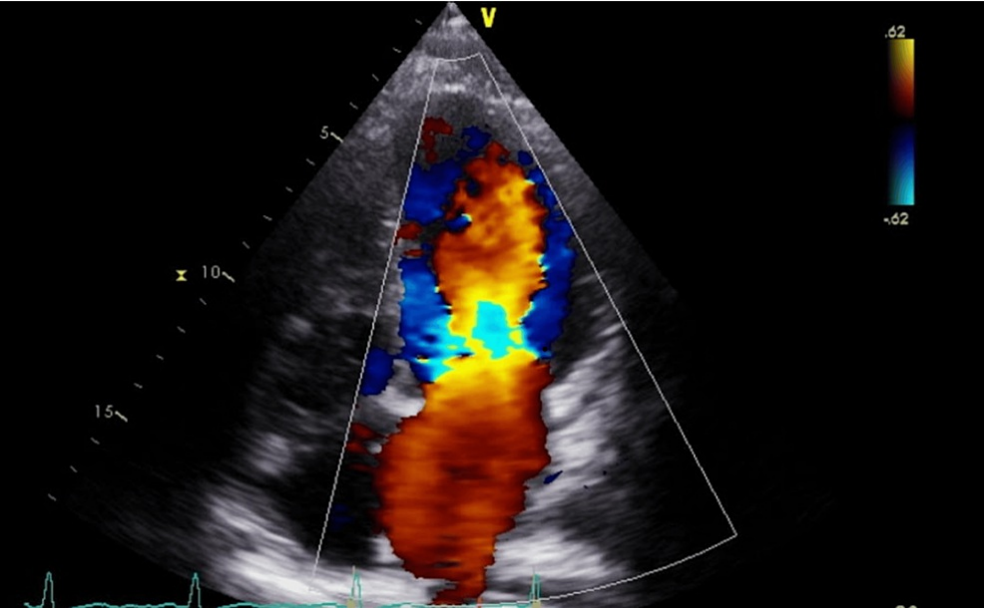
Echocardiogram Doppler visually demonstrates moderate to severe mitral regurgitation

On evaluation in the clinic, she appeared to be well-nourished. Her vitals revealed a blood pressure of 140/99 mmHg, a heart rate of 91, a SpO2 of 99% on room air, a respiratory rate of 16, and a body mass index of 40.2. On further questioning, the episodes appeared to be exclusively postcoital in nature. The duration and frequency of the episodes were not known. However, episodes did not occur while she was pregnant, and she abstained from coitus during pregnancy. There was no relationship between the hemoptysis episodes and her menstrual cycle. She also did not provide any history suggestive of vasculitis or connective tissue disease. Serological testing for vasculitis and connective tissue disease was unremarkable. Upon review of echocardiogram images, it was noticed that, although the MR velocity was not high, she visually appeared to have significant mitral regurgitation on Doppler imaging (Figure 2).

**Figure 3:**
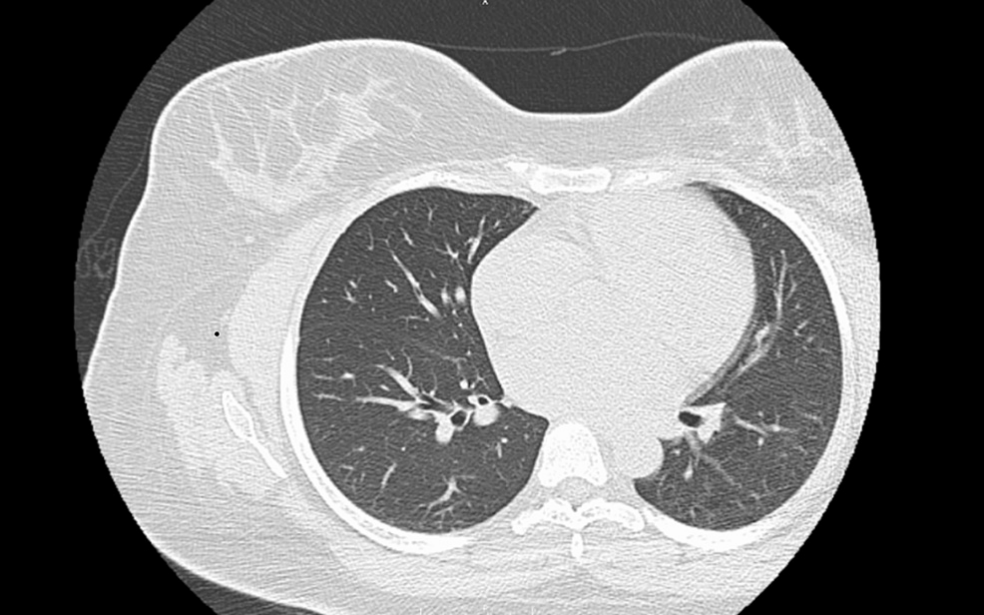
Computed tomography of the chest without contrast portrays complete resolution of the bilateral ground-glass opacities

The patient underwent a transesophageal echocardiogram, which confirmed malcoaptation of the mitral valve, causing moderate to severe mitral regurgitation. She established care with cardiology and was started on lisinopril 10 mg q.d. to control blood pressure and reduce afterload. She was advised to monitor her blood pressure and to watch her daily sodium intake. Since treatment, she has not experienced a similar episode. A follow-up CT scan showed the resolution of the ground glass opacities (Figure 3).

## Discussion

Exercise-induced pulmonary hemorrhage, commonly presenting as epistaxis, is a well-known phenomenon occurring in most breeds of racehorses after competitive events [2]. It is most likely due to the sharp rise in pulmonary artery wedge pressures and pulmonary capillary pressures that accompany the marked increase in cardiac output, sometimes exceeding 30 L/min, during vigorous exercise. At pulmonary capillary pressures exceeding 40 mmHg, alveolar capillaries rupture due to stress failure, causing hemorrhagic lung edema in the horse [3]. Exercise-induced hemoptysis is uncommon in humans, however, and post-coital hemoptysis is still rare.

Our patient’s postcoital hemoptysis can be attributed to the mitral regurgitation revealed on the transesophageal echocardiogram. Given that coitus is a form of mild-moderate exercise, the hemodynamic changes during both physical activities are comparable [4]. Both static and dynamic exercise are associated with the transient functional deterioration of mitral regurgitation. Although static exercise is not associated with a decrease in total peripheral resistance (and might even increase it), an increase in heart rate due to increased sympathetic activity causes the cardiac output to rise, imposing a pressure load on the left ventricle. Dynamic exercise, on the other hand, causes a volume load due to the increased venous return and reduced afterload, mediated by the peripheral venous skeletal muscle pump and arteriolar vasodilation. Typically, both the volume loading during dynamic exercise and the pressure loading during static exercise lead to exercise-induced ventricular dilation, impaired closure of the mitral valve cusps, dynamic worsening of mitral regurgitation, and reduced forward stroke volume. In addition, sympathetic activity during exercise increases the heart rate, causing decreased diastolic filling time and decreased outflow from the left atrium [5].

In healthy individuals, exercise is associated with only mild increases in left atrial pressure and mean pulmonary pressure. However, the combined effects of decreased outflow from the left atrium and more severe mitral regurgitation during coitus cause an exaggerated rise in left atrial pressures and pulmonary capillary pressures, predisposing patients to pulmonary congestion, pulmonary capillary rupture, and hemorrhage. This explains the hemoptysis noted clinically in our patient and the pulmonary edema seen on her chest X-ray.

An important question is why this patient, akin to the majority of patients in other case reports, did not experience hemoptysis following other exertional activities. Both coitus and other exertional activities lead to increased sympathetic tone, but what about sexual activity makes hemoptysis more likely? One proposed mechanism is the horizontal positioning commonly seen during sex. When positioned horizontally, there is less recruitment of the blood vessels in the upper lung fields because perfusion is already more symmetrical among all three lung fields due to gravity. The recruitment of fewer blood vessels means that the lung is less capable of accommodating the increased pulmonary blood flow during exercise and will further distend the perfused pulmonary vessels, increasing the risk of congestion and hemorrhage [6].

In addition to a cardiac workup, it is imperative to rule out pulmonary pathologies in a patient presenting with post-coital hemoptysis. Pulmonary infections, bronchial carcinoma, bronchiectasis, tuberculosis, and vasculitides are among the most common etiologies of hemoptysis in general. The work-up can include inflammatory markers, autoantibodies, CT chest and CT angiography, and bronchoscopy [7].

## Conclusions

Postcoital hemoptysis is a rare condition, usually associated with an underlying cardiovascular etiology, as previously reported in the literature. Adequate control of cardiovascular risk factors guides management and has been shown to improve outcomes. With this case report, we hope to contribute to the existing literature on hemoptysis associated with coitus.
